# The Clone Wars: Diagnosing and Treating Dysproteinemic Kidney Disease in the Modern Era

**DOI:** 10.3390/jcm10081633

**Published:** 2021-04-12

**Authors:** Rupali S. Avasare, Nicole K. Andeen, Andrea Havasi, Jonathan J. Hogan

**Affiliations:** 1 Division of Nephrology, Department of Medicine, Oregon Health & Science University, Portland, OR 97239, USA; avasare@ohsu.edu; 2 Department of Pathology, Oregon Health & Science University, Portland, OR 97239, USA; andeen@ohsu.edu; 3 Renal Section and Amyloidosis Center, Department of Medicine, Boston University School of Medicine, Boston, MA 02118, USA; ahavasi@bu.edu; 4 Division of Nephrology, Perelman School of Medicine, University of Pennsylvania, 3400 Spruce Street, Philadelphia, PA 19104, USA

**Keywords:** glomerular disease, dysproteinemia, monoclonal gammopathy, amyloidosis

## Abstract

Dysproteinemic kidney diseases are disorders that occur as the result of lymphoproliferative (B cell or plasma cell) disorders that cause kidney damage via production of nephrotoxic monoclonal immunoglobulins or their components. These monoclonal immunoglobulins have individual physiochemical characteristics that confer specific nephrotoxic properties. There has been increased recognition and revised characterization of these disorders in the last decade, and in some cases, there have been substantial advances in disease understanding and treatments, which has translated to improved patient outcomes. These disorders still present challenges to nephrologists and patients, since they are rare, and the field of hematology is rapidly changing with the introduction of novel testing and treatment strategies. In this review, we will discuss the clinical presentation, kidney biopsy features, hematologic characteristics and treatment of dysproteinemic kidney diseases.

## 1. Introduction

Dysproteinemic kidney diseases represent a spectrum of conditions caused by lymphoproliferative disorders that produce nephrotoxic monoclonal immunoglobulins. There have been significant advances in the understanding, diagnosis and treatment of dysproteinemic kidney disease in the last two decades. Before discussing the individual dysproteinemic kidney diseases in this review, it is useful to highlight some important terms and concepts [1].

- **Monoclonal gammopathy of undetermined significance (MGUS)** is a hematologic condition defined by presence of a monoclonal gammopathy in the serum at concentration of <30 g/L and <10% monoclonal plasma cells in the bone marrow, and the absence of organ damage from the monoclonal gammopathy.
- **Monoclonal gammopathy of renal significance (MGRS)** is a group of B cell or plasma cell clonal proliferative disorders that do not meet criteria for overt hematologic malignancy, but which produce monoclonal immunoglobulins that are toxic to the kidney. **The kidney biopsy (histologic) diagnoses are referred to as MGRS-associated disorders, lesions or conditions in patients who do not have overt malignancy.** This is a subtle, but important, difference in terminology that serves as a reminder that the essential step in managing dysproteinemic kidney is proper characterization and treatment of underlying hematologic (MGRS) disorder.
- **The majority of dysproteinemic kidney diseases exhibit monotypic staining on biopsy**, demonstrating that the monoclonal gammopathy is causing kidney damage. As discussed below, there are some exceptions, such as in cases of monoclonal gammopathy-associated C3 glomerulopathy and monoclonal gammopathy-associated thrombotic microangiopathy.
- **A comprehensive hematologic workup in collaboration with a hematologist is required once a dysproteinemic kidney disease is diagnosed**. This involves testing to find the underlying clone, which often includes bone marrow biopsy and aspirate, peripheral blood flow cytometry, and imaging such as positive emission tomography/computed tomography to search for evidence of extramedullary disease. It also involves characterizing dysproteinemia with serum and urine protein electrophoresis (sPEP, uPEP), serum and urine immunofixation (sIFE, uIFE), and the serum free light chain assay (sFLC).
- **The goal in treating dysproteinemic kidney disease is to eradicate the underlying lymphoproliferative disorder** that is producing the nephrotoxic paraprotein. This is true regardless of whether the underlying hematologic process is MGRS or overt malignancy. These treatments are often referred to as clone-directed, anti-plasma cell, or anti-B cell therapies since they are specific to clone type.
- **The evidence for the effectiveness of treatment is known as hematologic response**—that is, improvement and/or normalization of paraprotein levels in the blood and urine. These hematologic response criteria have been validated in multiple myeloma and AL (light chain) amyloidosis. In other MGRS-associated disorders, the myeloma and AL amyloidosis hematologic response criteria are often used to analyze response, but these criteria have not been systematically validated in these disorders.
- **Organ (kidney, heart, etc.) response is contingent on achieving a hematologic response.** Therefore, monitoring these hematologic labs in addition to kidney parameters is essential to gauge prognosis and the expectation of kidney response, as the organ response occurs after hematologic response.
- **Recurrent dysproteinemic kidney disease after kidney transplant is well-described in MGRS-associated disorders.** There are emerging data on optimizing outcomes for patients with end stage kidney disease due to dysproteinemic kidney disease undergoing kidney transplant, as well as the safety and efficacy of using newer clone-directed therapies in the setting of solid organ transplantation.

## 2. The Kidney Biopsy in Dysproteinemic Kidney Disease

Pathologically, paraprotein-driven kidney diseases often manifest as deposition of the monoclonal protein within glomeruli, tubulointerstitium, and/or vasculature (Table 1).

Deposits may be amyloidogenic (in immunoglobulin light chain (AL), heavy chain (AH) or heavy and light chain (AHL) amyloidosis), conventional immune complexes (for example, in proliferative glomerulonephritis with monoclonal immunoglobulin deposits (PGNMID)), have an organized tubular or microtubular substructure (for example, with immunotactoid glomerulopathy (ITG) and occasionally in cryoglobulinemic glomerulonephritis), or a fine “powdery” composition (as occurs in monoclonal immunoglobulin deposition disease (MIDD)). Filtered monoclonal proteins can also lead to tubular injury by altering cellular function or forming intracellular crystals, as occurs in light chain proximal tubulopathy (LCPT), or due to intratubular concentration in the distal nephron, as occurs in light chain cast nephropathy (LCCN). Although mechanisms are incompletely understood, physiochemical properties of monoclonal proteins and their specific interactions along the nephron—rather than sheer abundance—play a pivotal role in determining which MGUS will become an MGRS.

While most MGRS-associated lesions can be diagnosed on kidney biopsy with traditional workup of light microscopy, immunofluorescence for immunoglobulins and complements on frozen tissue (IF-F), and electron microscopy, contemporary studies have highlighted the importance of additional techniques specifically IgG subclass staining, paraffin immunofluorescence (IF-P), and DNAJB9 immunohistochemistry in certain settings. IgG subclass staining is utilized to demonstrate IgG heavy chain restriction in addition to light chain restriction [21], particularly for type I cryoglobulinemic glomerulonephritis, PGNMID, and IgG heavy chain deposition disease. IF-P is required to reveal (or exclude) “masked” monoclonal proteins in some cases of C3-dominant glomerulonephritis [17], and in many cases of crystalline LCPT [22,23]. The etiology of “masked” deposits is unknown but potentially related to hidden or altered epitopes, interactions with IF transport media, or limitations of laboratory antibodies to react with epitopes across the great biologic repertoire of immunoglobulins. In contrast, both IgG subclasses and IF-P may be also be required to reveal an oligo- or polyclonal process which appears to be monoclonal by traditional methods. Specifically, most cases of “monoclonal” fibrillary glomerulonephritis are polyclonal or re-classifiable after additional evaluation with IgG subclass stains, IF-P, and DNAJB9 [24].

In addition to injury from direct deposition and filtration of paraproteins, indirect effects of monoclonal proteins and lymphoproliferative disorders on the kidney may be seen. Data to support C3 glomerulopathy in the setting of a monoclonal gammopathy represent an MGRS-associated lesion comes from epidemiologic data [25,26,27], elegant in vitro case studies [18,19,28], and outcome studies [20,29,30]. Thrombotic microangiopathy (TMA) has infrequently been associated with paraproteinemia [31,32].

## 3. Light Chain Cast Nephropathy

**Figure 1:**
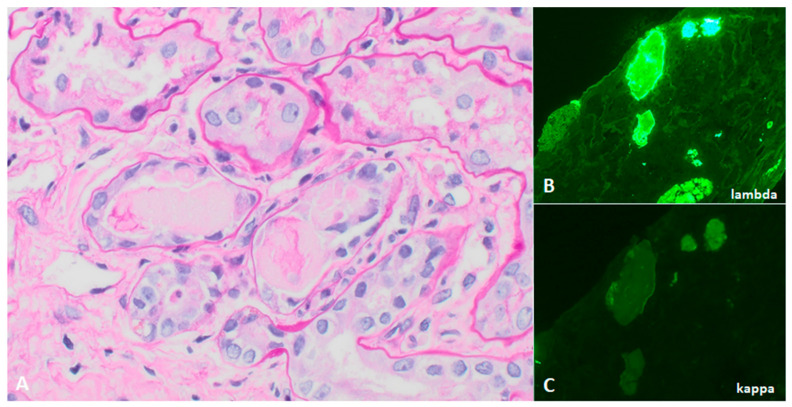
Light chain cast nephropathy, with (**A**) PAS-pale, fractured tubular casts with a cellular reaction (PAS 400×) which stain for (**B**) lambda light chain on immunofluorescence microscopy; (**C**) kappa light chain is negative in casts.

Light chain cast nephropathy (LCCN) is the most common form of kidney injury in multiple myeloma and is found in up to 35% of kidney biopsies from multiple myeloma patients [33,34]. Risk factors for LCCN include older age, high serum monoclonal light chain burden, volume depletion, hypercalcemia, and diuretic-use [33]. Patients are often asymptomatic until the late stages of kidney disease, and disease may be incidentally discovered on blood and urine tests that show abnormal serum creatinine, non-albumin proteinuria (i.e Bence-Jones proteinuria), +/− hematuria. Kidney biopsy is recommended in patients with albuminuria (>10% of total proteinuria) because this suggests glomerular involvement, which is more commonly seen in other paraprotein-related diseases, such as MIDD and amyloidosis [34]. Kidney biopsy is usually deferred when there is a high clinical suspicion of LCCN, specifically when the involved serum light chain exceeds 500 mg/L in patients with predominantly non-albumin proteinuria [35,36]. The hallmark biopsy finding in LCNN is tubular casts with a “hard” or “fractured” appearance, which stain pale on periodic acid-Schiff or polychromatic on trichrome. These casts are composed of monoclonal light chains and uromodulin. Their aggregation in the distal tubule elicits a giant cell reaction with associated acute tubular injury and tubulointerstitial inflammation [37]. There is bright staining for one light chain by immunofluorescence (Figure 1).

Molecular interactions between uromodulin (at free light chain-binding domain, D8C) and a hypervariable segment on the free light chain (CDR3) are influenced by amino acid residues in the CDR3 region [2,3]. These structural interactions are determinants of cast formation, and can be inhibited by competitor peptides in a rodent model of LCCN, highlighting the importance of interventions which increase intratubular flow, in addition to chemotherapy [2,3]. The extent of cast formation, and tubular atrophy and interstitial fibrosis have been associated with renal outcome [38]. Light chain casts may also show amyloid-like properties, with birefringence on Congo Red stain under polarized light; this intratubular amyloid in LCCN has been associated with systemic AL amyloidosis [39]. LCCN is considered a myeloma-defining event, but may infrequently be seen in the setting of Waldenström macroglobulinemia and CLL [40].

Initial therapy is centered around correcting volume depletion, hypercalcemia and decreasing light chain production [41]. Anti-myeloma therapy in the current age is highly effective at decreasing light chain production. The combination of a proteosome inhibitor and dexamethasone +/− an immunomodulatory drug or cyclophosphamide is now first-line therapy for most patients, with daratumumab-lenalidomide-dexamethasone being an alternative first line regimen. Plasma exchange removes light chains, though most studies investigating its use were done prior to widespread use of proteosome-inhibitors and failed to show clear and consistent benefit [42]. Thus, plasma exchange is now reserved for unique circumstances [43,44]. Two recent randomized trials evaluated high cut-off (HCo) dialysis versus conventional hemodialysis for dialysis-dependent AKI from LCCN but neither achieved the primary end-point of reduced dialysis independence at 3 months, however the MYRE trial suggested a possible benefit for HCO-dialysis at the 6 and 12-month time point [45,46]. Rapid light chain reduction is associated with improved kidney outcomes, including reversal of kidney injury and dialysis-discontinuation [47,48]. Older age, lower initial kidney function, higher baseline light chain level, and higher B2-microglobulin (partially due to diminished renal clearance) levels were associated with worse kidney function during follow-up [38]. Reassuringly, as therapies have improved, the proportion of end-stage kidney disease patients with multiple myeloma has declined and rates of kidney recovery have improved [49,50]. Furthermore, transplantation is an option for select patients in remission (very good partial or better) from cytogenetically standard-risk multiple myeloma [51,52].

## 4. Light Chain Proximal Tubulopathy

Light chain proximal tubulopathy (LCPT) accounts for 4% of biopsied light chain-related kidney disease, and >80% of these patients have MGRS [4,53,54]. The pathogenic light chains accumulate in proximal tubule (PT) lysosomes and disrupt the cell’s resorptive capabilities, resulting in renal Fanconi syndrome [55]. These tubular light chain inclusions may be crystalline or non-crystalline, and kappa has been more commonly implicated than lambda light chain for crystalline variants [4]. Patients present with complete or incomplete Fanconi syndrome (hypokalemia, hypophosphatemia, aminoaciduria, normoglycemic glycosuria and elevated fractional excretion of uric acid and phosphorus) and slowly progressive chronic kidney disease that can result in kidney failure. Other clinical features include osteomalcia from chronic hypophosphatemia. Disease is diagnosed by serum and urinary findings consistent with Fanconi syndrome or by kidney biopsy [56].

**Figure 2:**
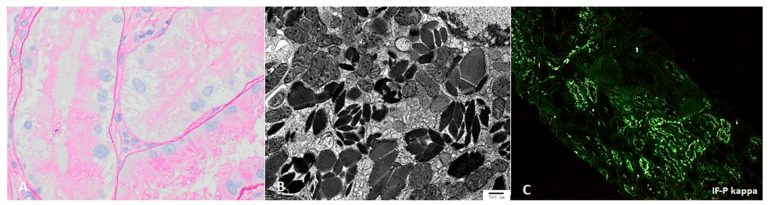
Light chain proximal tubulopathy, with rhomboid-shaped intratubular crystals ((**A**) PAS 600× and (**B**) transmission EM, direct mag 14,000×), which stain for (**C**) only kappa light chain, by paraffin-immunofluorescence.

Light chain proximal tubulopathy (LCPT) has crystalline or non-crystalline inclusions of monoclonal light chains in proximal tubules with associated tubular injury; similar crystalline paraprotein inclusions can be identified in other kidney cells, including podocytes [57,58] and histiocytes [4,59,60] (Figure 2).

Like other proteins, these are physiologically reabsorbed via the megalin/cubulin pathway in proximal tubules, but properties of the variable (V) region in particular confer increased resistance to proteolysis or degradation by lysosomal enzymes, and propensity for crystal formation [4,5,6]. This intercellular accumulation of monoclonal free light causes increased oxidative stress and apoptosis [2]. The majority of crystalline LCPT is kappa light chain, and non-crystalline LCPT is generally lambda light chain [4,53]. IF-P is substantially more sensitive than IF-F for identifying crystalline LCPT [4]; since light microscopic findings may also be subtle, electron microscopy and IF-P can be critical for identifying LCPT [53,61]. In approximately 75% of patients with LCPT, diagnosis on renal biopsy represents the initial identification of a lymphoproliferative disorder [4,53].

Disease recognition is important because treatment can stabilize kidney disease. Several treatments have been described in the literature, ranging from monitoring alone to autologous stem cell transplant. Treatment that induces a hematologic response is associated with improved kidney outcomes [4].

## 5. AL (Light Chain) Amyloidosis

Up to 85% of renal amyloidosis are immunoglobulin-derived, 75% of which are light chain (AL) lambda [62]. In AL amyloidosis, end organ damage is primarily caused by aggregation and progressive deposition of immunoglobulin light chain or light chain fragments leading to disruption in the local tissue structure; however, the light chains themselves have also been shown to be cytotoxic (see LCPT above) [63,64,65,66]. Heavy chain (AH) and heavy/light-chain amyloidosis (AHL) are rare entities in which the amyloid deposits are derived from fragments of the heavy chain only or from both heavy chain and light chain, respectively [67]. Approaches to diagnosis, treatment and outcome in AH and AHL amyloidosis are similar to AL amyloidosis. In spite of substantial progress in improving patient outcomes with AL amyloidosis, the exact mechanism of fibril formation remains unknown.

**Figure 3:**
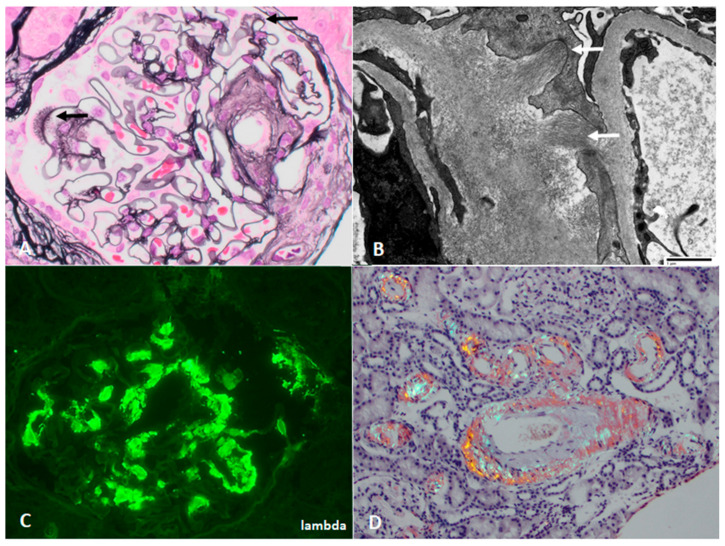
Kidney biopsy images of AL amyloidosis, with (**A**) “spicules” on Jones stain (arrows, 600×) and (**B**) haphazardly arranged fibrils infiltrating capillary loops with associated podocyte foot process effacement (arrows, transmission EM direct mag 6800×), corresponding with the common nephrotic presentation. Deposits show (**C**) smudgy glomerular staining for lambda light chain, and (**D**) prominent vascular involvement is also present (Congo red stain under polarized light, 200×).

The diagnosis of amyloidosis requires a tissue biopsy showing the presence of amyloid fibrils. On light micrograph, amyloid most commonly forms amorphous deposits in the glomerular, tubulointerstitial, and/or vascular compartment, which are pale when stained with H&E and weakly positive with periodic acid-Schiff (PAS). As a result of the unique beta pleated sheet configuration, amyloid deposits show characteristic apple green birefringence under polarized light when stained with Congo red or yellow-green fluorescence with Thioflavin T [68]. The randomly arrayed, non-branching fibrils are 7–12 nanometers in diameter on electron micrograph (Figure 3).

Amyloid typing, to confirm AL type, is critical before treatment recommendations can be made. Typing by immunofluorescence microscopy is highly sensitive for AL amyloidosis, and shows bright, smudgy staining restricted to one light chain. In challenging cases, immunogold electron microscopy or liquid chromatography–tandem mass spectrometry on laser dissected deposits can be performed to confirm amyloid type [69].

The localization of the amyloid deposits affects the clinical presentation. All organs, except the central nervous system, can be affected in AL amyloidosis, leading to a variety of symptoms and signs. Most of the symptoms of AL amyloidosis are not specific, frequently leading to late diagnosis. The kidneys are involved in 70–80% of cases [70,71,72] and it most commonly manifests as high-grade proteinuria and/or deteriorating renal function due to glomerular deposition. However, there are exceptions to this rule: sometimes proteinuria is non-existent or only minimal, as in cases where the deposits are small during the early stage of the disease process or when the deposits are found mainly in the vessels or in the interstitium.

In AL amyloidosis the plasma cell burden is usually low, but 5–10% of the cases are associated with multiple myeloma or Waldenström macroglobulinemia. Rarely, it can also be the consequence of LC production by non-Hodgkin lymphoma with variable clone burden. A monoclonal protein is detectable in serum and/or urine in >98% percent of patients by using the combination of serum and urine immunofixation along with a serum free light chain (FLC) assay [13].

Treatment of AL amyloidosis targets the abnormal plasma cell clone in order to halt the production of amyloidogenic light chains. According to the response criteria described by Palladini et al. [73], complete hematologic response (CR) is defined as negative serum and urine protein electrophoresis and immunofixation, as well as normal serum free light chain (FLC) ratio; very good partial response (VGPR) is defined as the difference between involved and uninvolved LCs (dFLC) < 40 mg/L [13]. Partial hematologic response (PR) is achieved when dFLC is decreased by >50% in patients with baseline dFLC > 50 mg/L and in some cases, it has also led to improved outcomes but the best overall survival is observed in CR followed by VGPR [69,74]. Interestingly, amyloid fibrils are very stable structures that can take years to resolve. By mass spectrometry, persistent amyloid deposits contain serum amyloid P (SAP) and apolipoprotein E, but show decreased amounts of Ig. With therapy, hematologic and renal response occur despite persistence of tissue amyloid deposits, perhaps due to eradication of amyloid protein oligomers or protofibrils which can have greater toxicity than mature fibrils [7,8,9,10].

Over the last decade renal staging and renal response criteria have been developed to predict the risk of end stage kidney disease in AL amyloidosis based on baseline kidney function and proteinuria (Stage I: estimated glomerular filtration rate (eGFR) ≥ 50 mL/min/1.73 m^2^ and <5 g/24 h proteinuria; Stage II: either eGFR < 50 mL/min/1.73 m^2^ or ≥5 g/24 h proteinuria; Stage III: eGFR < 50 mL/min/1.73 m^2^ and ≥5 g/24 h proteinuria) [65,70,75]. At 6 or 12 months post-treatment, the cutoff for predicting favorable kidney outcome with therapy was a ~30% decrease in proteinuria (or a drop of proteinuria below 0.5 g/24 h) without worsening of eGFR, while a ~25% decrease in eGFR correlated with worse renal outcome [73,75].

Survival has improved considerably in the last two decades due to the rapidly expanding arsenal of effective therapies for achieving deep and durable hematologic responses [69,76,77,78,79]. These therapies include proteasome inhibitors (bortezomib, carfilzomib, ixazomib), immunomodulatory drugs (pomalidomide, lenalidomide, thalidomide) and monoclonal antibodies (daratumumab, elotuzumab, isatuximab) [80,81,82]. The most aggressive form of treatment is high-dose melphalan with autologous stem cell transplantation (HDM/SCT) but due to concerns about treatment-related morbidity and mortality, only 20% of newly diagnosed patients with AL amyloidosis are eligible for, and eventually undergo, this form of therapy [70,83]. A rapid decrease in the circulating LC concentration in patients who achieve a hematologic CR or VGPR can reverse proteinuria and renal dysfunction over time with median time from hematologic response to renal response of ~10–11 months [73,75,84]. Unfortunately, some patients do not achieve renal organ response despite experiencing hematologic response.

Concurrent with the improvement in overall patient survival, there is increasing incidence of chronic kidney disease leading to end stage kidney disease in patients with AL amyloidosis (14–42% of AL patients with kidney involvement) [70,85]. Recent studies demonstrated good kidney transplant outcomes in a selected group of patients who achieved hematologic CR or VGPR with anti-plasma cell treatments and who did not have significant extra-renal amyloid involvement [86]. Additionally, second- and third-line treatments have been successfully deployed in kidney transplant recipients after hematologic relapse. Determining eligibility criteria and management of AL amyloidosis in the setting of kidney transplantation requires a multidisciplinary approach involving experienced nephrologists, transplant surgeons and hematologists [86].

## 6. Type I and Type II Cryoglobulinemic Glomerulonephritis

Cryoglobulins (CGs) are immunoglobulins or their fragments that precipitate below 37 °C and are classified by clonality. Type I involve complexes of monoclonal immunoglobulins and type II is a monoclonal immunoglobulin against a polyclonal immunoglobulin. Type III CGs are complexes of polyclonal immunoglobulins and therefore are not considered dysproteinemias [87].

Type I CGs are almost exclusively due to lymphoproliferative disorders. In two recent large series of type I cryoglobulinemia MGUS was the culprit in up to 40% of cases, and IgG was the predominant involved Ig [88,89]. In contrast to Type 1 IgG CGs, Type 1 IgM CGs are less likely due to MGUS, and more likely due to Waldenström macroglobulinemia (lymphoplasmacytic lymphoma). Type 1 CG causes a small vessel vasculitis and as such, cutaneous manifestations are present in most patients, while kidney involvement was found in up to 30% of patients [88,89,90].

The guiding principle for treatment is finding and targeting the causative clone. A thorough hematologic malignancy workup that may include a bone marrow aspirate and biopsy, peripheral blood flow cytometry, and appropriate imaging is necessary to delineate the underlying lymphoproliferative process. A variety of clone-directed treatments, ranging from rituximab to bortezomib-based multi-chemotherapy regimens, to stem cell transplant, have been described in the literature. Kidney response to treatment is as high as 85%, though many patients require repeated rounds of therapy with multiple agents and relapse is common [88,89,90]. Plasma exchange has been used for rapidly progressive disease with end-organ damage. However, the strongest indication for this is hyperviscosity [91] syndrome.

Type II CGs are due to hepatitis C virus infection in 60–90% of cases with the remaining due to autoimmune diseases (30% of non-infectious cases), and lymphoproliferative disorders (21% of non-infectious cases) [92]. When the underlying cause is unidentified, as is the case in approximately 50% of non-infectious Type II CG, the condition is termed essential mixed cryoglobulinemia. The top lymphoproliferative disorders associated with Type II CG are marginal zone lymphoma, low-grade non-Hodgkins B-cell lymphoma and lymphoplasmacytic lymphoma [92]. The classic Meltzer’s triad of purpura, arthralgias and weakness is seen in up to 30% of patients [93]. Kidney involvement is present in 35% of patients and approximately 1/3 of patients present with decreased GFR. Kidney findings include hematuria, proteinuria, kidney insufficiency and hypertension. In addition to an abnormal paraprotein workup, other laboratory abnormalities include low serum complement levels (often C4), elevated rheumatoid factor, and a high cryocrit, though importantly, the cryocrit percentage does not always correlate with disease severity and treatment response [94].

Similar to Type I CG, the treatment is targeted to the underlying disease. Rituximab therapy is associated with favorable outcomes in the overall Type II CG population, however the risk of infection was nine-fold higher with this therapy [94]. Similar to Type I CG, overall remission of kidney disease has been reported in up to 85% of patients [92]. Plasma exchange is reserved for those with life-threatening disease, including rapidly progressive glomerulonephritis.

**Figure 4:**
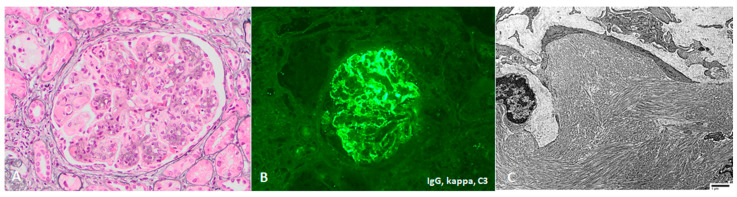
Cryoglobulinemic glomerulonephritis, with (**A**) a membranoproliferative pattern of injury and intracapillary cryoglobulin “plugs” (Jones 200×), (**B**) segmentally accentuated mesangial and capillary staining for monotypic IgG and C3 by IF, and (**C**) organized microtubular substructure by EM (direct mag 2900×).

On kidney biopsy, cryoglobulinemic GN (Figure 4) has an endocapillary to membranoproliferative pattern of injury, with influx of monocytes and neutrophils. Large intracapillary “plugs” of immune complexes may be present, as well as extraglomerular leukocytoclastic vasculitis. Immunofluorescence shows irregularly distributed, chunky capillary wall staining for the monoclonal protein in type 1 cryoGN, commonly IgG and one light chain, and the addition of IgM in type 2 cryoGN. Electron microscopy shows occasionally large subendothelial and mesangial deposits, some with microtubular organized substructure.

## 7. Monoclonal Immunoglobulin Deposition Disease

**Figure 5:**
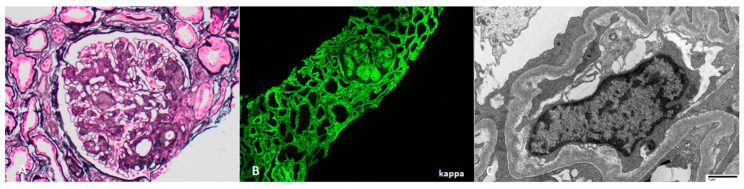
Monoclonal immunoglobulin deposition disease, with (**A**) a nodular glomerulopathy (Jones 200×), (**B**) diffuse staining of glomeruli, tubular basement membranes, and interstitium, usually for one light or heavy chain by immunofluorescence, and (**C**) punctate “gunpowder” immune deposits by EM (direct mag 4800×).

Monoclonal immunoglobulin deposition disease (MIDD) (Figure 5) may be light chain (LCDD), heavy chain (HCDD), or both (LHCDD), and is characterized by a diabetes-like glomerulosclerosis with thickened tubular basement membranes by light microscopy, widespread tubular basement membrane and glomerular staining for the monoclonal Ig by immunofluorescence (Figure 5), and fine “powdery” subendothelial and tubular immune deposits by electron microscopy [95].

Approximately 80% of LCDD are kappa light chain, and characteristics of the light chain hypervariable region (CDR) have been linked to pathogenicity [11]. Investigators have proposed that the mechanism of LCDD may be related to hydrophobic amino acid residues or glycosylation which modify the light chain conformation and interactions with cells and matrix [11,12]. Notably, N-glycosylation makes the monoclonal protein less detectable in historical clinical testing [11], but has been associated with progression of plasma cell disorders in more advanced assays [96]. HCDD is most commonly IgG heavy chain, but IgA and rarely IgM and IgD HCDD have been described [13]. In contrast to LCDD, IgG HCDD has the consistent finding of a truncated of heavy chain, with deletion in constant domain 1 (C_H_1) which can produce disease in mouse models [12,13,14,15].

Patients with MIDD present with kidney insufficiency, often with nephrotic-range proteinuria (more common in HCDD than LCDD), high blood pressure, and an abnormal paraprotein workup, including an abnormal serum and urine IFE in 90% of patients [97]. A large French series described extrarenal manifestations in approximately 35% of patients, most commonly involving the heart and liver, and less commonly the lungs [97,98]. The underlying hematologic disorder in MIDD is MGRS in up to 65% of cases, and is associated with multiple myeloma in the rest [97]. In one retrospective review of patients from the Mayo Clinic, 63% of those who did not receive specific therapy progressed to ESKD versus 38% of those who had either chemotherapy and/or stem cell transplant [95]. Another review of patients from the United Kingdom with exclusively LCDD showed that most patients who achieved a hematologic CR or VGPR did not progress to kidney failure, whereas patients who did not respond to chemotherapy progressed to end stage kidney disease [99]. A French study showed the rate of response to chemotherapy was similar across MIDD subtypes whether or not it was associated with multiple myeloma [97]. Kidney transplantation may be considered in those who have VGPR or CR because recurrent disease leading to graft loss in these select patients appears to be low [97,99,100].

## 8. Immunotactoid Glomerulopathy

**Figure 6:**
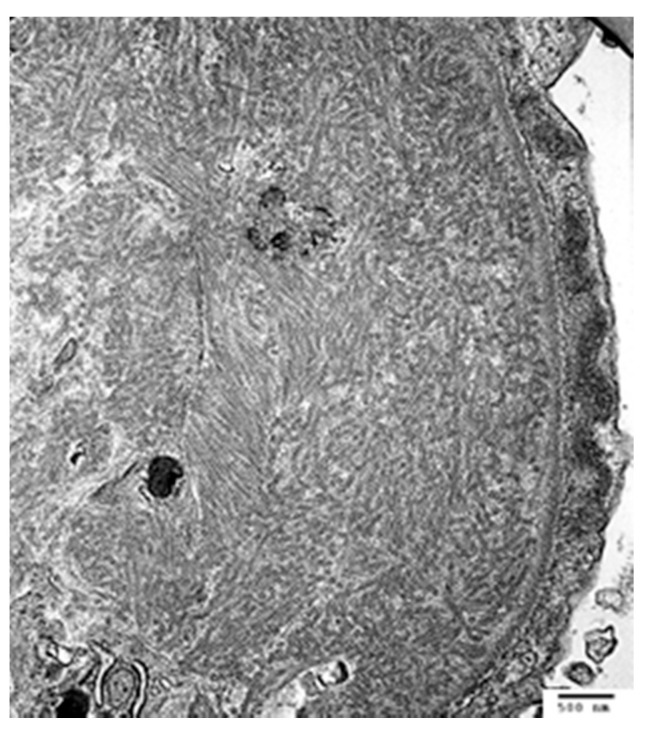
Immunotactoid glomerulopathy, with monoclonal Ig deposits with large microtubular substructure, hollow cores, arranged in focally parallel arrays (mesangial deposits, direct mag 18,000×).

Immunotactoid glomerulopathy (ITG) is seen in less than 0.1% of kidney biopsies and has an endocapillary proliferative, membranoproliferative, or membranous pattern of injury by light microscopy [101]. Two thirds of cases are monoclonal, which are usually composed of IgG1 (67%) and kappa (63%), with none showing IgG3 restriction in the largest series, in contrast to PGNMID [101]. By electron microscopy (Figure 6), immune deposits of ITG have a predominantly microtubular substructure with hollow cores arranged at least focally in parallel arrays, with a median diameter of 28 nm [101]. The average fibril diameter less than 20 nm in 22% of cases, which may have diagnostic overlap with the fibril size of fibrillary glomerulonephritis (with fibrils 12–24 nm) [101], but these can be distinguished by the consistent expression of DNAJB9 which is restricted to fibrillary glomerulonephritis [102,103,104]. Cryoglobulinemic glomerulonephritis may also have morphologic similarities, but patients with ITG have negative serum cryoglobulins and lack the extra-renal involvement that is common in cryoglobulinemia [101].

The description of ITG had been described in small case series until recently, when two publications significantly enhanced the characterization of ITG comprising 100 cases in total [101,105]. The first study was published by the Mayo Clinic and Columbia University Renal Pathology Groups and described 73 cases of ITG, 67% of which exhibited monotypic staining, and 33% of which exhibited polytypic staining. The second publication was a multi-center collaboration in France of 27 patients with monoclonal ITG only. IgG kappa was present in 63% of cases vs. IgG lambda in 37%. Paraprotein and clone detection rates were high for monoclonal ITG patients in both studies.

From a hematologic perspective, monoclonal ITG is unique among dysproteinemic kidney diseases in that the underlying hematologic disorder is often a B cell clone, as opposed to most others where plasma cell clones are most common. Indeed, the French group, which performed bone marrow biopsy in all patients, found a lymphoproliferative disorders in the majority of cases, and these B cell clones had cell surface markers consistent with those found in chronic lymphocytic leukemia (CLL) [105]. The use of clone-directed therapy was associated with improved kidney outcomes in both studies, and the French study showed that achieving sustained hematologic response (by CLL criteria in patients with B cell clones and myeloma criteria for patients with plasma cell clones) was associated with improved kidney outcomes. Thus, it is clear that patients with monoclonal ITG should undergo a full hematologic workup with a specific focus on assessing for B cell clones and receive clone-directed therapy with the goal of achieving a hematologic response to improve kidney outcomes.

The approach to management for patients with polyclonal ITG is less clear. One-quarter of polyclonal ITG cases in the Columbia-Mayo Clinic study had an underlying lymphoproliferative disorder, which is antithetical to current practice in MGRS-associated disorders, since a full hematologic workup is initiated only after detecting monotypic staining on kidney biopsy [101]. These patients require further study.

## 9. Proliferative Glomerulonephritis with Monoclonal Immunoglobulin Deposits

**Figure 7:**
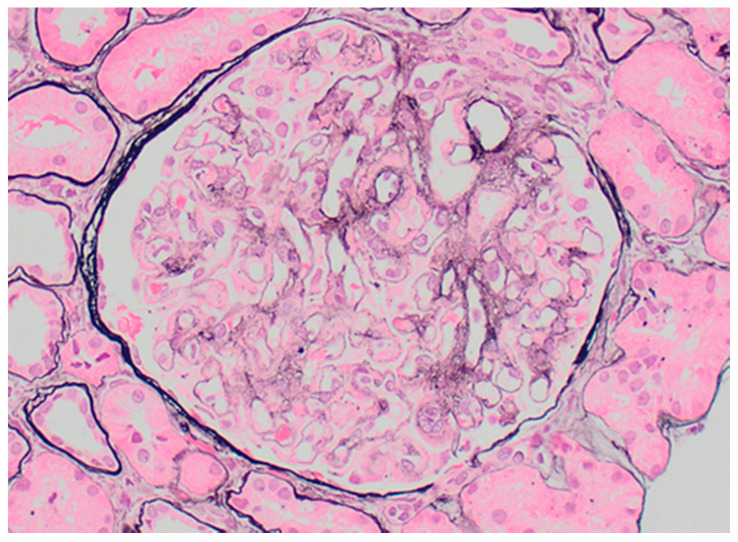
Proliferative glomerulonephritis with monoclonal deposits, with prominent mesangial and capillary wall eosinophilic immune deposits (Jones 200×).

Proliferative glomerulonephritis with monoclonal immunoglobulin deposits (PGNMID-Ig) is defined on kidney biopsy as light microscopy findings of proliferative or membranoproliferative glomerulonephritis (Figure 7), with immunofluorescence or immunohistochemistry showing monotypic glomerular deposits for a heavy chain (IgG, IgA, or IGM) and an associated light chain (kappa or lambda), and electron microscopic findings of granular, glomerular electron dense deposits [106,107,108]. The lack of substructure on electron microscopy distinguishes PGNMID-Ig from immunotactoid glomerulopathy and cryoglobulinemic glomerulonephritis. IgG3 is the most common involved IgG subclass (60–70% of cases, usually with kappa light chain), followed by IgG1, IgG2 and IgG4. Cases of monotypic staining involving IgM and IgA heavy chains are also well-recognized.

PGNMID-Ig presents most commonly in patients aged 50–70 with kidney insufficiency and proteinuria, often in the nephrotic range, and sometimes with overt nephrotic syndrome. PGNMID-Ig also has rarely been reported in children [109,110,111]. Historically, the renal prognosis for patients with PGNMID-Ig has been poor.

On hematologic workup, the percentage of patients with detectable dysproteinemia and underlying clonal proliferative disorders is low, ranging from 25 to 40%, with no clear predilection for clone-type (i.e., plasma cell, B cell or lymphoplasmacytic clone) [107,112,113,114]. The low rate of paraprotein and clone detection in PGNMID presents challenges since the majority of patients cannot have true clone-directed therapy, and also lack paraprotein studies for evidence of hematologic response. The reason for this low rate of clone and paraprotein detection is not clear. The definitions of hematologic and renal response criteria also require development and validation in PGNMID-Ig, as most available literature have used criteria extrapolated from other glomerular and/or hematologic disorders.

While the data are limited, the use of clone-directed therapy in patients with detectable clones is consistent with well-established practice in other dysproteinemic kidney diseases, and may lead to high rates of renal response. The treatment of patients who do not have a detectable clone is controversial. Some experts advocate for the use of empiric therapy with agents that would target the hypothesized clone, as opposed to non-specific immunosuppression that is not routinely used for lymphoproliferative disorders (such as mycophenolate mofetil), and there are some data supporting improved renal outcomes with this strategy [114]. There has been enthusiasm around using daratumumab as an empiric agent for PGNMID, but this requires study and more detailed characterization of the underlying lymphoproliferative disorder.

Recurrence of PGNMID after kidney transplant is common. One recent case series of 26 patients who found an 89% recurrence rate of disease at a mean of 5.5 months after transplant. The overall median graft survival was 92 months, but 11/26 patients lost their graft due to PGNMID at mean of 36 months after transplant [115]. One recent case series described improved outcomes of post-transplant PGNMID treated with rituximab (*n* = 9) compared to cyclophosphamide with or without plasmapheresis (*n* = 4) [116]. The interpretation of the results of both of these studies are limited by lack of full characterization of the underlying clonal disorder, which may impact approach to treatment and kidney outcomes.

## 10. Light Chain Variant of Proliferative Glomerulonephritis with Monoclonal Immunoglobulin Deposits

A recently published multicenter case series describes the clinicopathologic characteristics of 17 patients with the light chain variant of PGNMID (PGNMID-LC), which, as the name suggests, shares the light and electron microscopic features of PGNMID-Ig, but is distinguished by immunofluorescence microscopy staining for kappa or lambda light chain only, without staining for IgG, IgM or IgG heavy chain [16]. In this series, the majority of these patients had kappa PGNMID-LC (71%), and the clinical presentation was similar to many dysproteinemic kidney diseases, as most patients presented in middle-age, with renal insufficiency and nephrotic range proteinuria. Hematologically, however, PGNMID-LC seems to be distinct from PGNMID-Ig, as most patients have both detectable paraproteinemia (sIFE 65%, uIFE 73%, abnormal sFLC assay 83%) and plasma cell clones detected on bone marrow biopsy (88%, with 71% diagnosed with MGRS and 29% diagnosed with multiple myeloma; plasmacytosis on bone marrow ranged from 2–90%). The best kidney outcomes were observed in patients who achieved complete hematologic response, most often due to clone-directed therapy with bortezomib-based therapy and/or autologous stem cell transplantation. Thus, PGNMID-LC is important to recognize due to the high rate of detecting an underlying clone and detectable dysproteinemia, which have implications for clinical management.

## 11. Monoclonal Gammopathy-Associated C3 Glomerulopathy

Monoclonal gammopathy-associated C3 Glomerulopathy (MG-C3G) is classified by the 2019 IKMG Consensus Report as a MGRS-associated disorder that does not exhibit monotypic deposits on kidney biopsy [1], with in vitro, observational and epidemiological evidence supporting the link between dysproteinemia and the development of C3G. There are two cases of patients with C3 glomerulopathy and lambda light chain monoclonal gammopathy that caused in vitro activation of the alternative complement pathway via binding to Factor H [18,19]. An epidemiologic study found a higher prevalence of monoclonal gammopathy in patients with C3G (30%) than in the general population, particularly in patients ≥50 years of age (65%) [30].

Pathologically, kidney biopsies exhibit a membranoproliferative pattern of injury by light microscopy, and C3 dominant staining as defined by the consensus report [117] with no or minimal staining for Ig by frozen IF [25,27,29,30]. In older adults, when considering a diagnosis of C3G, IF-P should be performed to exclude “masked” monoclonal Ig deposits [17]. The mechanism for false-negative staining by routine IF is not known, but approximately 36–50% of apparent C3G in adults with monoclonal gammopathy may have “masked” Ig deposits, which is a different entity and pathogenesis, although both may be related to an underlying lymphoproliferative disorder [17]. In C3G, ultrastructural studies demonstrate mesangial, subendothelial, intramembranous and subepithelial deposits, or ultradense “sausage-shaped” intramembranous deposits in dense deposit disease (DDD). The distinction between C3G and DDD is based on electron microscopy studies; they share clinical, pathologic, and mechanistic features, although the incidence of “masked” Ig deposits is much higher in C3G than DDD [17].

There is also evidence from the French National Database of C3G that improved kidney outcomes may be achieved using a hematologic approach to diagnosis and of MG-C3G. This study carefully characterized 50 patients with MG-C3G with regard to hematologic diagnosis (60% MGRS, 34% multiple myeloma, 6% chronic lymphocytic leukemia) and complement pathway studies. Treatment with clone-directed therapy, mainly with bortezomib-based regimens, and achieving a complete or very good hematologic response (using AL amyloidosis hematologic response criteria) was associated with superior kidney outcomes.

Given these data and the lack of definitive treatment strategies for C3G, it is advisable to screen all patients with C3G for the presence of monoclonal gammopathy, with particular attention paid to patients over the age of 50. In patients with MG-CG3, a full hematologic workup and consideration of clone-directed therapy is warranted, with the goal of achieving a complete or very good hematologic response.

## 12. Monoclonal Gammopathy-Associated Thrombotic Microangiopathy

Monoclonal gammopathy-associated thrombotic microangiopathy has been classified by the 2019 IKMG Consensus Report as a provisional MGRS-associated lesion [1], and similar to MG-associated C3 glomerulopathy, is a dysproteinemic kidney disease that does not exhibit monotypic staining on kidney biopsy. The data supporting a biological association between monoclonal gammopathies and the development of thrombotic microangiopathy are based on clinical observations and epidemiologic data [32,118,119]. Illustrative cases include a patient who did not respond to eculizumab but who had a hematologic and renal response with treatment against a small plasma cell clone with bortezomib-lenalidomide-dexamethasone [32], and cases of patients with myeloma who developed thrombotic thrombocytopenic purpura associated with anti-ADAMTS13 antibodies [120,121]. There is also evidence that patients with thrombotic microangiopathy on kidney biopsy have a higher prevalence of monoclonal gammopathy than the general population after adjustment for age [119]. The pathogenesis of thrombotic microangiopathy in the setting of monoclonal gammopathy is not clear, but has been hypothesized to involve immunoglobulin-associated endothelial injury, anti-ADAMTS13 antibodies, alternative complement pathway activation and hyperviscosity.

## Figures and Tables

**Table 1:** Kidney biopsy characteristics of dysproteinemic kidney diseases.

	Light Microscopy	Frozen IF Microscopy	Electron Microscopy	Additional Studies/Notes	Postulated Mechanism(s) of Kidney Injury
Light chain cast nephropathy	Hard, fractured casts, giant cell reaction, ATI, interstitial inflammation	Cast staining for one light chain	Not specific	Can be diagnosed with IF-P if necessary	Interactions between Tamm-Horsfall protein and hypervariable region of light chain, tubular microenvironment [2,3]
Light chain proximal tubulopathy	Acute tubular injury, crystalline tubular inclusions	Good sensitivity for lambda, poor sensitivity for kappa/crystalline deposits	Tubular crystals or prominent enlarged lysosomes	IF-P often required for crystalline variant	V region changes which confer resistance to proteolysis and/or aggregation and crystal formation [2,4,5,6]
AL amyloidosis	Acellular deposits in glomeruli, vessels, tubulointerstitium	Bright, smudgy staining for one Ig, often lambda	Infiltrative fibrils 7–12 nm	Congo red positive.	Deposits of misfolded immunoglobulin, serum amyloid P, and apolipoprotein E; certain Ig sequences have been associated with amyloidogenic properties [7,8,9,10]
Monoclonal immunoglobulin deposition disease	Nodular mesangial sclerosis and thickening of tubular basement membranes	Bright staining of tubular basement membranes and glomeruli for monoclonal Ig, often kappa	Fine, punctate non-fibrillar deposits	IgG subclasses can confirm IgG HCDD.	LCDD: amino acid properties in hypervariable region, including hydrophobicity and glycosylation [11,12] IgG HCDD: deletion of constant domain 1 (C_H_1) [12,13,14,15]
Immunotactoid glomerulopathy	Endocapillary to MPGN pattern, membranous	66% monoclonal, often IgG1 kappa	Fibrils with hollow cores and parallel arrays, ~30 nm	Exclude cryoglobulinemia. IgG subclasses confirm IgG monoclonality. DNAJB9 negative	Unknown
Cryoglobulinemic glomerulonephritis	Endocapillary to MPGN pattern	Glomerular staining for IgG, IgM, and light chain(s)	Subendothelial, mesangial deposits, some with microtubular organized substructure	IgG subclasses confirm IgG monoclonality. Substructural organization in isolation is insensitive to identify cryoGN.	Type I: temperature and concentration-dependent aggregation of monoclonal protein, with small vessel occlusion and injury Type II: Monoclonal IgM directed against Fc portion of polyclonal IgG
PGNMID-Ig	Endocapillary to MPGN pattern	Glomerular staining for one IgG heavy and light chain (usually IgG3 kappa), C3	No substructural organization	IgG subclasses confirm IgG monoclonality. Exclude cryoglobulinemia.	Unknown
PGNMID-Light chain variant	Endocapillary to MPGN pattern	Glomerular staining for one light chain (often kappa) and C3	Subendothelial, mesangial, subepithelial immune deposits [16]		Unknown
MG-associated C3 Glomerulopathy and Dense Deposit Disease (DDD)	MPGN	C3 only or dominant staining; exclude masked deposits	Subendothelial and mesangial deposits; in DDD, ultradense intramembranous deposits	IF-P to exclude masked monoclonal deposits [17]	Monoclonal protein causes dysregulation of alternative complement pathway [18,19,20]

**Abbreviations:** IF—immunofluorescence microscopy; ATI- acute tubular injury; IF-P—immunofluorescence microscopy performed on paraffin embedded tissue; HCDD—heavy chain deposition disease; LCDD—light chain deposition disease; MPGN- membranoproliferative glomerulonephritis; PGNMID—proliferative glomerulonephritis with monoclonal immunoglobulin deposits; MG—monoclonal gammopathy; AL—light chain.

## Data Availability

Not applicable.
